# From needs to design: teachers' perspectives on core components of culturally responsive SEL in Türkiye

**DOI:** 10.3389/fpsyg.2026.1814578

**Published:** 2026-03-31

**Authors:** Melek Alemdar, Mehmet Aşıkcan

**Affiliations:** 1 Manchester Institute of Education, The University of Manchester, Manchester, United Kingdom; 2 Ahmet Keleşoglu Education Faculty, Necmettin Erbakan University, Konya, Türkiye

**Keywords:** culturally responsive education, needs analysis, primary education, qualitative research, social and emotional learning (SEL), thematic analysis (TA), Türkiye

## Abstract

Social and emotional learning (SEL) is increasingly recognized as culturally situated rather than universally uniform. In Türkiye, SEL was integrated into the national curriculum for the first time in 2024 through the Türkiye Century Education Model (TCEM). Yet this reform was launched without a systematic needs assessment grounded in teachers' perspectives, despite teachers being the primary actors who encounter students' social, emotional, and behavioral challenges in everyday classroom life. This study aimed to examine how Turkish primary school teachers conceptualize students' SEL needs and to interpret these understandings within a culturally responsive framework. A qualitative design was employed, with 12 focus groups involving teachers from 26 cities across Türkiye (*N* = 171). Data were analyzed through reflexive thematic analysis, supported by strategies of credibility, dependability, and confirmability. The findings reveal that teachers view SEL not as abstract skill lists but as interdependent capacities enacted within daily school practices. Four interconnected clusters of need were consistently prioritized: (1) emotional awareness, expression, and regulation; (2) communication, cooperation, and adaptation; (3) self-regulation, responsibility, and problem-solving; and (4) empathy, compassion, and respect. Teachers further underscored systemic preconditions for effective SEL, including teacher capacity, alignment between home and school expectations, and whole-school coherence. The study contributes to global SEL scholarship by offering contextualized evidence from a non-Western setting and by demonstrating how teachers actively negotiate between universal SEL discourses and local educational realities. Implications are discussed for the design of culturally responsive SEL curricula, teacher professional development, and policy initiatives in comparable contexts.

## 1 Introduction

Social and emotional competencies are widely recognized as central to children's mental wellbeing, school engagement, and long-term developmental outcomes (Goodman et al., 2015), and substantial evidence shows that these skills are both malleable and teachable (Jones and Kahn, 2017). Consequently, social and emotional learning (SEL) has gained strong international momentum and is often described as “the soul” or “missing piece” of contemporary education systems (Elias et al., 1997; Humphrey, 2013). In Türkiye, scholars similarly argue that an exclusive emphasis on cognitive performance is insufficient for preparing children for social and labor-market demands, underscoring the need for explicit and systematic SEL provision [Agirkan and Ergene, 2022; Esen-Aygün, 2017; Kaplan and Göl-Güven, 2026; Turkish Industry and Business Association (TÜSIAD), 2019].

These discussions have become increasingly salient given Türkiye's complex socio-demographic landscape, shaped by socio-economic inequality, large refugee and migrant populations, recent earthquakes, and substantial regional and cultural diversity (Alemdar, 2025). Such conditions influence children's daily experiences of stress, belonging, and relational life, making culturally responsive SEL not merely desirable but necessary. In response to evolving educational needs, the Turkish Ministry of National Education (MEB) introduced the Türkiye Century Education Model (TCEM) and the accompanying K-12 Skills Framework, which—for the first time—embed SEL explicitly within a national curriculum (Aşkar and Altun, 2023; Özhan et al., 2023). TCEM defines SEL as

“*the acquisition and use of the knowledge, skills and tendencies necessary for individuals to manage their emotions, empathise, build supportive relationships and develop a healthy sense of self in accordance with personal and social goals*,” [Ministry of National Education (MEB), 2023, p. 38]

an understanding that shows clear conceptual alignment with Collaborative for Academic, Social, and Emotional Learning (CASEL). However, TCEM's SEL structure was developed without a systematic needs assessment with teachers, the practitioners who observe children's social, emotional, and behavioral challenges most directly. While this reform represents an important policy step, it also raises critical questions in a culturally diverse, partly collectivist, Middle Eastern context (Hofstede, 2011; Imamoglu and Gültekin-Yasak, 1993): To what extent is the model culturally grounded, and to what extent does it replicate Western, individualistic SEL assumptions? In this study, culturally responsive SEL refers to the alignment between SEL frameworks and the relational norms, communication practices, and social expectations that shape everyday classroom life in Türkiye. From this perspective, SEL competencies may be broadly shared across contexts, yet their meanings and classroom enactment are shaped by locally embedded expectations regarding emotion, communication, and responsibility. Given shifting student demographics and the educational needs of children, understanding this alignment is essential.

This study addresses these concerns through a multi-site qualitative needs assessment involving 171 primary school teachers across Türkiye. It examines which SEL competencies teachers perceive as most lacking, which competencies and implementation conditions they view as essential for a future SEL curriculum, and how these articulated needs align with the SEL competencies embedded in TCEM.

## 2 Literature review

### 2.1 Social and emotional learning

SEL is commonly defined as “an integral part of education and human development” that enables individuals to understand and manage emotions, demonstrate empathy, build relationships, make responsible decisions, and navigate social contexts [Collaborative for Academic, Social, and Emotional Learning (CASEL), 2025; Jones et al., 2021]. A substantial and increasingly global evidence base shows that well-implemented SEL programmes strengthen intrapersonal and interpersonal skills [Organisation for Economic Co-operation and Development (OECD), 2021; Wigelsworth et al., 2016], enhance mental health and emotional wellbeing (Dray et al., 2017), and reduce bullying, victimization, and violence in school settings (Downes and Cefai, 2016). Beyond these developmental benefits, SEL is consistently associated with improved classroom behavior, more positive peer relationships, and enhanced academic performance, as well as longer-term outcomes including higher graduation rates, reduced antisocial behavior, and increased employability (Albright et al., 2019; Cipriano et al., 2023; Ha et al., 2025).

Because SEL is inherently ecological, scholars emphasize that schools play a central role in shaping children's emotional and social development through curriculum design, pedagogical practices, school climate, and whole-school approaches (Humphrey et al., 2025). For these reasons, SEL has become a central pillar of educational reform worldwide, with increasing attention to how programmes can be adapted and sustained across diverse cultural and socio-political contexts.

### 2.2 Western-origin SEL frameworks: the OECD Big Five model and CASEL

Frameworks serve as organizing structures that guide goal setting, curriculum development, assessment, and communication in SEL implementation (Blyth et al., 2018). Among the most influential frameworks shaping contemporary SEL policy are the OECD Big Five Social and Emotional Skills model and the framework developed by the CASEL. Although originating from different disciplinary traditions, both models reflect a Western empirical lineage and offer convergent conceptualisations of core social–emotional competencies.

The OECD Big Five model is grounded in personality psychology and synthesizes decades of empirical research into five broad domains: conscientiousness, extraversion, agreeableness, emotional stability, and openness to experience. Developed through lexical analyses of natural language descriptors, the model provides a parsimonious and empirically robust structure for summarizing social and emotional characteristics (Chernyshenko et al., 2018).

Parallel to this, CASEL has played a defining role in translating social–emotional constructs into school-based practice (Dusenbury and Weissberg, 2018). CASEL conceptualizes SEL through five interrelated competencies—self-awareness, self-management, social awareness, relationship skills, and responsible decision-making—which integrate cognitive, emotional, and behavioral capacities central to learning and development [Collaborative for Academic, Social, and Emotional Learning (CASEL), 2025; Weissberg et al., 2015]. While emerging from educational and prevention science rather than personality psychology, CASEL's framework aligns closely with the regulatory, interpersonal, and self-related dimensions articulated in the Big Five model.

Together, these two frameworks are among those that have shaped global understandings of SEL as a set of malleable, developmentally sensitive competencies that support both academic learning and broader life outcomes [Ministry of National Education (MEB), 2023]. At the same time, scholars have raised concerns regarding how such frameworks—developed largely within Western epistemological traditions—translate into culturally and institutionally diverse contexts, particularly with respect to relational norms, moral priorities, and implementation processes (Jones et al., 2019; Jukes, 2019; Mahfouz and Anthony-Stevens, 2020). These concerns underscore the importance of examining not only conceptual alignment but also how universal SEL frameworks are interpreted and enacted within specific educational systems.

### 2.3 SEL in Türkiye and the TCEM

In response to global shifts toward whole-child education, Türkiye has recently introduced the TCEM, representing the country's first nationwide and systematic effort to integrate SEL skills into the national curriculum. TCEM does not position SEL as a standalone programme or subject area; instead, it conceptualizes social and emotional competencies as transversal capacities intended to permeate teaching and learning across the entire K-12 system.

Policy documents indicate that TCEM draws heavily on internationally established SEL frameworks, particularly the OECD's Big Five Social and Emotional Skills model and the CASEL framework [Ministry of National Education (MEB), 2023]. Consistent with these models, SEL within TCEM is broadly defined as a set of competencies that enable individuals to understand themselves, regulate emotions, establish positive relationships, demonstrate empathy, adapt to changing contexts, and make responsible decisions [Ministry of National Education (MEB), 2024].

Within TCEM, SEL competencies are organized into three overarching domains: self-skills, social life skills, and compound skills (see Table 1). These domains broadly correspond to intrapersonal, interpersonal, and integrative competencies and are operationalised through detailed process indicators that describe how skills are expected to develop across learning experiences. Self-skills encompass self-awareness, self-regulation, and self-reflection; social life skills include communication, collaboration, and social awareness; and compound skills—situated at the intersection of self and social domains—comprise adaptability, flexibility, and responsible decision-making. In this respect, TCEM aligns closely with dominant Western-origin SEL frameworks.

**Table 1:** Social-emotional learning skills and process indicators.

SEL domain	Social-emotional skills	Process indicators
Self skills: refer to being aware of one's own characteristics and the reflections of these characteristics on emotions, thoughts, and behaviors.	Self-awareness	• Identifying how to learn a new topic/concept or information. • Recognizing the emotions experienced in response to events/situations. • Engaging in activities to increase awareness of one's own emotions.
	Self-regulation	• Setting goals to meet one's own needs. • Adjusting motivation. • Monitoring and managing one's own emotions, thoughts, and behaviors to achieve a goal. • Making self-assessment in the process of achieving a goal. • Engaging in activities to improve one's learning situation.
	Self-reflection	• Evaluating oneself. • Transforming emotions/thoughts/behaviors.
Social life skills: refer to the skills individuals use to actively participate in social life and to meet the challenges they face in their daily lives.	Communication	• Active listening. • Expressing feelings and thoughts. • Engaging in verbal or non-verbal interaction. • Taking part in group communication. • Removing communication barriers.
	Collaboration	• Collaborating with individuals and groups. • Discussing/negotiating ideas with others. • Reconciling differing opinions and building partnerships. • Taking part in team work and cooperation. • Implementing outcomes from social interactions.
	Social awareness	• Paying attention to social cues. • Understanding other people's feelings, thoughts and perspectives. • Developing understanding and respect for others. • Developing an understanding of social norms.
Compound skills: refer to the skills individuals use to actively participate in social life and to meet the challenges they face in their daily lives.	Adaptability	• Understanding new, changing and uncertain situations. • To be open-minded and willing to change in the face of new, changing and uncertain situations. • Adapting mindsets for new, changing and uncertain situations. • Adapting ways, levels and degrees of behavior or action under new, changing and uncertain circumstances. • Managing emotional responses to interact successfully with new, changing and uncertain environments.
	Flexibility	• Finding alternative solutions to difficult situations. • Adapting to new situations.
	Responsible decision-making	• Identifying problems and how to solve them. • Anticipate the consequences of actions. • Making reasoned decisions. • Evaluate the ethical appropriateness of decisions taken. • In-depth thinking.

While the architecture of the programme reflects an ambitious and holistic vision of education, it also introduces a critical tension between conceptual breadth and pedagogical operability. Although TCEM delineates SEL domains and accompanying process indicators, these elements are not consistently translated into subject-level learning outcomes, instructional guidance, or assessment practices. In a highly centralized system where textbooks effectively function as the curriculum, such indeterminacy raises questions about how SEL is to be made visible, coherent, and consistent across classrooms and regions.

Rather than positioning SEL as a standalone strand, TCEM situates social–emotional competencies within a network of interrelated components, including the *Virtue–Value–Action framework*—articulating nationally endorsed values such as justice, mercy, responsibility, and respect—alongside *systems thinking and literacy skills*. In addition, TCEM introduces dispositions as another core component of the model. The dispositions (e.g., empathy, curiosity, self-confidence, trust, questioning, etc.) are conceptualized as relatively stable identity-related, social, and intellectual tendencies that motivate and activate skills across contexts, shaping how learners engage with content, peers, and challenges rather than being taught through direct instruction [Ministry of National Education (MEB), 2024].

Moreover, although TCEM foregrounds social interaction and cognitive regulation, emotional and identity-related competencies—such as belonging and compassion—remain weakly articulated within the SEL strand itself and are instead distributed across the aforementioned components of the model. This structural dispersion reflects an implicit assumption that such competencies will emerge organically through participation, values education, or dispositions, rather than through sequenced, active, focused, and explicit (SAFE) instructional practices (Durlak et al., 2011; Greenberg, 2023).

Taken together, TCEM reflects a significant policy alignment with global SEL movements while also highlighting the challenges of implementing universal frameworks within socio-culturally complex contexts. These tensions point to the importance of examining teachers' perspectives, as teachers are the primary implementers of SEL and are well-positioned to identify how social and emotional competencies are understood and supported in everyday classroom practice.

### 2.4 Culturally responsive SEL

Teaching and learning are deeply embedded in culture and shaped by culturally grounded norms of emotion, communication, relational obligation, and social responsibility (Parrish and Linder-VanBerschot, 2010). Because SEL is inherently concerned with how individuals understand themselves, relate to others, and regulate behavior within social contexts, questions of culture are not peripheral but central to SEL design and implementation. A critical issue, therefore, concerns whose cultural knowledge, values, and interactional norms are reflected in SEL frameworks (Lipka et al., 2005).

A growing body of scholarship highlights that many widely adopted SEL models, including those informing international policy agendas, originate primarily in Western, educated, industrialized, rich contexts (Jones et al., 2019; Mahfouz and Anthony-Stevens, 2020). Frameworks such as CASEL and the OECD's Big Five model emphasize competencies that align closely with socio-emotional norms prioritized in the United States and similar contexts, including individual self-regulation, verbal self-expression, and proactive interpersonal engagement. While these competencies are empirically robust and broadly relevant, their meanings, behavioral expressions, and social functions may vary substantially across sociocultural settings (Brush et al., 2022; Demkowicz et al., 2026).

Importantly, cultural misalignment does not necessarily stem from the skills themselves, but from how those skills are defined, taught, and enacted in everyday practice. For example, emotional competence may be expressed through verbal articulation in some cultural contexts, while restraint, indirect communication, or relational sensitivity may signal maturity and social competence in others. Similarly, responsibility and autonomy may be enacted through individual initiative or through relational obligation, depending on cultural norms (Chen and Yu, 2022). These variations underscore that SEL effectiveness depends not only on what skills are targeted, but on how those skills resonate with local meanings and lived experiences.

Evidence from the Turkish context further illustrates how cultural norms shape emotional expression and regulation. Cross-cultural research on emotion socialization suggests that Turkish parents tend to encourage children's expression of negative emotions less frequently and engage less in explicit discussion of emotions compared with parents in the United States, reflecting cultural expectations emphasizing obedience, relational harmony, and the regulation of behaviors that might disrupt group cohesion (Kiliç et al., 2026). At the same time, Turkish parents demonstrate relatively higher encouragement of children's positive emotional expression, indicating that emotional socialization practices are not uniformly restrictive but calibrated according to culturally embedded display rules. Such findings suggest that children may enter classrooms with emotion repertoires shaped by norms that privilege relational attunement, restraint, and context-sensitive emotional expression.

This continuity becomes particularly visible when Western-originated communicative frameworks are introduced into Turkish educational settings without contextual adaptation. For example, counseling trainees in Türkiye who are taught Rogers' and Carkhuff's micro-skills are often expected to use frequent verbal reflection of feelings and content—an interactional style that, as Keklik (2023, p. 130) notes, “often leads to discomfort in clients because they are not used to such ways of communication.” In this sense, what is pedagogically coded as competent practice—explicit emotional articulation and sustained verbal engagement—may sit uneasily with locally familiar ways of relating, where restraint, indirectness, and relational attunement can index social maturity and respect. Thus, students may be required not only to acquire new skills but also to suspend established communicative repertoires and adopt unfamiliar interactional norms, raising concerns about cultural fit and “cultural safety” when such practices are transferred without contextual integration (Keklik, 2023). While this example is drawn from counselor education, it points to a broader dynamic: what counts as competent social–emotional practice is not self-evident but culturally negotiated.

These considerations suggest that evaluating SEL frameworks solely at the level of competency definitions may obscure how these competencies are interpreted, enacted, and prioritized in everyday educational practice. Understanding teachers' situated perspectives therefore becomes critical for assessing whether SEL models resonate with the relational norms and pedagogical realities of specific educational contexts.

In response to these concerns, scholars have increasingly argued for culturally responsive approaches to SEL—approaches that do not merely adapt surface features of existing frameworks but actively engage with the cultural contexts in which SEL is implemented (Jukes, 2019). In this study, culturally responsive SEL refers to the alignment between SEL frameworks and the lived social realities, relational expectations, and pedagogical practices of the contexts in which they are enacted. Within the Turkish educational context, this perspective emphasizes how competencies such as emotional regulation, communication, responsibility, and empathy are interpreted and practiced within culturally embedded interactional norms. Culture is therefore treated not as a variable to be adjusted after implementation, but as the context through which SEL becomes meaningful, teachable, and sustainable in everyday classroom life.

### 2.5 The present study

Within nationally standardized education systems such as Türkiye's, implementing SEL poses distinctive challenges. Although the TCEM draws on internationally established SEL frameworks [Ministry of National Education (MEB), 2023], it is enacted within a socio-culturally heterogeneous context shaped by regional diversity, migration, and socioeconomic inequality (Alemdar, 2025). In such contexts, teachers function as key cultural mediators of SEL, interpreting curricular expectations and translating them into everyday classroom practice. Engaging with teachers' lived experiences, therefore, provides a critical lens for assessing the contextual relevance and practical feasibility of SEL frameworks, as well as for identifying how research-based knowledge can be meaningfully translated into educational practice (Solberg et al., 2020). Also, centring practitioner perspectives is particularly important for ensuring that educational research remains grounded in real-world conditions and responsive to implementation challenges (Biesta, 2007). Primary school teachers observe students' social–emotional strengths and difficulties on a daily basis and bear primary responsibility for enacting SEL within highly structured, curriculum-driven environments. Their perspectives thus offer valuable insight into both students' immediate needs and the systemic conditions that shape SEL implementation.

Building on evidence that SEL initiatives are more effective and culturally responsive when implementers' contextual knowledge and perceived needs are systematically examined (Gay, 2021; Graves et al., 2017), the present study adopts a qualitative needs assessment approach centered on teachers' perspectives. Despite the recent integration of SEL into the national curriculum through TCEM, no published multi-site qualitative study has examined which SEL competencies primary school teachers perceive as most urgently needed, nor how these perceived needs align with the competencies articulated in the national framework.

To address this gap, the present study is guided by the following research questions:

- Which social–emotional competencies do primary school teachers perceive as the most pressing needs among their current students?
- According to teachers, which implementation conditions should be prioritized in the development of an effective and contextually responsive SEL curriculum?
- To what extent do teachers' articulated SEL needs align with the competencies outlined in the TCEM?

## 3 Method

This study employed a qualitative, interpretive design to examine how primary school teachers in Türkiye understand, prioritize, and make sense of SEL within their everyday classroom practice. This orientation is consistent with interpretive and phenomenological approaches that foreground meaning-making as it occurs in context, through direct engagement with practice (Giorgi and Giorgi, 2003; Moustakas, 1994).

An interpretive (hermeneutic) phenomenological stance guided the inquiry, emphasizing how teachers' understandings of SEL are constituted through culturally, historically, and institutionally embedded experiences rather than abstract definitions (Heidegger, 1927/1962; van Manen, 2023). In this sense, the study aligns with applied phenomenological work in education that uses teachers' accounts to illuminate how pedagogical meaning is constructed *in situ* (Laverty, 2003).

Teachers' accounts were interpreted with explicit attention to the socio-cultural and structural features of schooling in Türkiye, including regional diversity, migration-related heterogeneity, and the constraints of a centralized, textbook-driven education system. Consistent with practice-oriented qualitative research, teachers' narratives were treated as situated pedagogical knowledge, reflecting how SEL is enacted, negotiated, and evaluated within everyday instructional and relational contexts rather than as decontextualised beliefs or attitudes (Biesta, 2007).

Within this interpretive framework, the researcher was understood as an active participant in the analytic process. Meaning was conceptualized as emerging through dialogue between participants' accounts, the researcher's theoretical sensibilities, and ongoing engagement with the literature (Gadamer, 1960/1989). Reflexivity was therefore treated not as a methodological limitation but as an analytic resource, supporting transparency in how interpretations were formed, questioned, and refined over time (Alsaigh and Coyne, 2021).

Data were analyzed using reflexive thematic analysis as an analytic strategy compatible with an interpretive phenomenological orientation (Braun and Clarke, 2019). Rather than functioning as a purely technical coding procedure, thematic analysis was used to identify patterned meanings across teachers' accounts while remaining grounded in descriptions of lived classroom experience (van Manen, 2023). This approach enabled the analysis to move beyond surface-level categorization of SEL skills and to capture the relational priorities, value orientations, and contextual logics shaping teachers' understandings of social–emotional competence in practice (Frechette et al., 2020; Smith et al., 2009).

### 3.1 Participants and sites

We recruited primary school teachers working across diverse geographical regions of Türkiye to capture variation in how SEL is experienced and interpreted in different educational contexts. We used purposeful sampling, a strategy commonly employed in qualitative research to select participants who have direct experience with the phenomenon under investigation and can provide information-rich accounts relevant to the research questions (Creswell and Poth, 2018; Patton, 2014; Yildirim and Simşek, 2021). Within this framework, we adopted maximum variation sampling to ensure diversity in regional location, school context, and years of professional experience.

We deliberately sought variation across urban, semi-urban, and rural school settings, as well as across schools serving socio-economically advantaged, disadvantaged, and mixed student populations. This sampling strategy enabled us to examine how SEL-related experiences and needs are shaped by differing socio-cultural, institutional, and regional conditions.

We organized participants into focus groups based on the provinces in which they worked. Grouping teachers by geographical proximity allowed us to preserve contextual similarity within each focus group and facilitated the emergence of shared experiential themes shaped by regional characteristics (e.g., Erzurum-Elazig-Sivas-Tokat-Konya; Izmir-Istanbul-Kütahya-Bilecik; Sirnak-Muş-Bingöl-Van). We labeled the 12 focus groups as FG1–FG12. Table 2 presents the distribution of participants by city clusters, the number of teachers in each group, and their ranges of teaching experience.

**Table 2:** Study group.

Code	City group	Number of teachers	Teaching experience (years range)	Interview duration (min)	Interview date
FG1	Erzurum–Elazig-Sivas-Tokat-Konya	9	5–23	49	25.08.2025
FG2	Izmir–Istanbul–Kütahya–Bilecik	26	3–30	96	27.08.2025
FG3	Manisa–Tekirdag	22	3–27	62	25.08.2025
FG4	Mugla–Aydin	15	5–20	58	26.08.2025
FG5	Nigde–Nevşehir	19	8–29	60	26.08.2025
FG6	Samsun–Ordu	21	8–26	70	26.08.2025
FG7	Sirnak–Muş–Bingöl-Van	8	8–23	49	25.08.2025
FG8	Sakarya-Kilis-Sirnak	11	6–29	53	27.08.2025
FG9	Düzce-Zonguldak	21	8–29	63	26.08.2025
FG10	Trabzon	19	7–34	65	25.08.2025
FG11	Tokat	10	8–25	43	25.08.2025
FG12	Zonguldak Izmir Istanbul	4	16–30	55	25.08.2025

**Figure 1:**
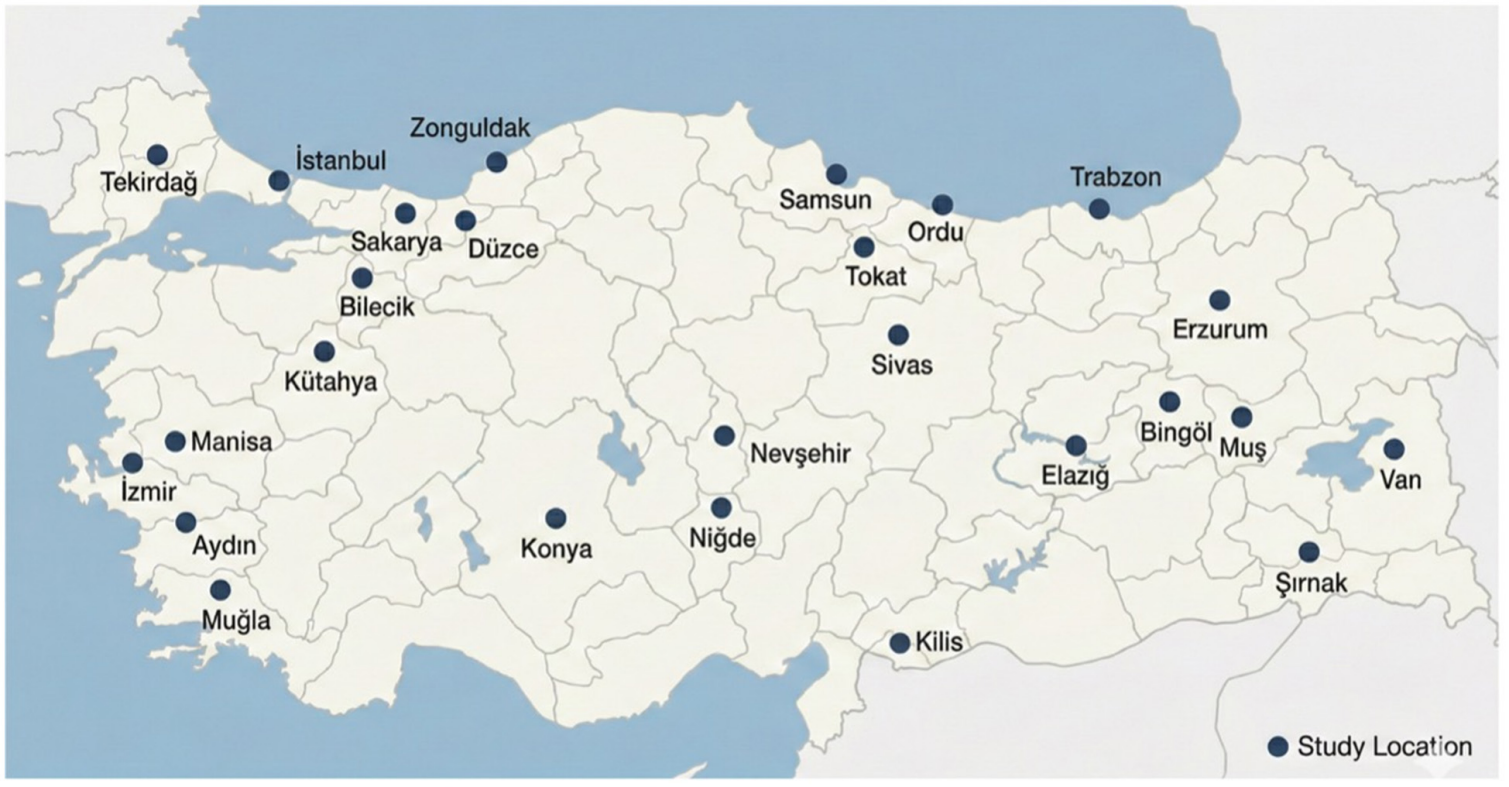
Geographic distribution of participating teachers across 26 cities in Türkiye.

As shown in Table 1 and Figure 1, the study group reflects wide geographical and professional diversity. The number of teachers in each focus group ranged from 4 to 26, and years of teaching experience ranged from 3 to 34. All participants were actively teaching Grades 1–4 at the time of data collection and had direct, ongoing experience with the social and emotional challenges faced by young learners.

### 3.2 Data collection

Data were collected through face-to-face focus group interviews with primary school teachers. Focus groups were selected because they provide an interactive setting in which participants can share experiences, negotiate meanings, and build on one another's reflections, generating rich and practice-oriented data on SEL (Kitzinger, 2005; Krueger and Casey, 2015). All focus groups followed a common semi-structured interview guide to ensure comparability across sites while allowing contextual elaboration (See OSF; https://osf.io/qwkth/files/23aub). The guide first elicited teachers' own understandings of SEL and subsequently explored how these understandings informed their perceptions of students' needs and curricular priorities. This sequencing enabled teachers' SEL conceptions to function as an analytic lens for interpreting student needs.

Interviews were conducted between 25 and 28 August 2025 in Ankara and were moderated by researchers with expertise in educational psychology, curriculum and instruction, and primary education. Moderators facilitated balanced participation, probed for clarification, and kept discussions focused on teachers' lived classroom experiences. A total of twelve focus group interviews were conducted, with durations ranging from 49 to 96 min. All sessions were audio-recorded with consent and transcribed verbatim in Turkish. Identifying information was removed during transcription and analysis, and pseudonyms were used throughout.

Data collection and preliminary analysis proceeded iteratively. Data saturation was considered achieved when additional focus group discussions no longer generated new themes or substantially different perspectives regarding students' social–emotional learning needs. In the later focus groups, teachers' accounts largely reiterated previously identified competency clusters and implementation concerns, suggesting that thematic saturation had been reached.

### 3.3 Data analysis

We analyzed the data using reflexive thematic analysis, supported primarily by NVivo 14. Reflexive thematic analysis is a flexible and theoretically informed qualitative approach that emphasizes sustained engagement with the data and treats meaning-making as an iterative and reflexive process rather than a linear coding exercise (Braun and Clarke, 2019). This approach is well-suited to phenomenological and interpretive research, as it enables the identification of patterned meanings while remaining sensitive to context, complexity, and researcher positionality.

The analytic process integrated inductive and deductive elements. Inductively, themes were grounded in teachers' own language, priorities, and lived experiences. Deductively, analysis was informed by the SEL literature and by the competency domains articulated in the TCEM. Two researchers independently coded the full dataset in NVivo. Coding proceeded through the following stages:

1. Familiarization: both coders read and re-read all transcripts to develop an in-depth understanding of the data, attending to recurring patterns as well as context-specific nuances.
2. Initial coding: we conducted line-by-line coding, generating codes that captured both explicit references (e.g., empathy, self-regulation, parental overinvolvement) and more implicit meanings (e.g., feeling unseen, silent compliance, peer cruelty). Coding remained open and expansive at this stage.
3. Developing candidate themes: codes were grouped into broader analytic categories (e.g., current needs of the students, emotion regulation challenges, family as facilitator or barrier, implementation conditions). Through iterative discussion and comparison across coders, these categories were refined into candidate themes.
4. Reviewing and finalizing themes: candidate themes were reviewed against the full dataset to ensure internal coherence and clear distinction between themes. Discrepancies between coders were resolved through discussion, leading to the finalization of four overarching themes, each comprising several subthemes. Theme names were selected to reflect teachers' own emphases while maintaining analytic clarity.
5. Linking themes to the policy framework: in a final analytic step, teachers' articulated needs and priorities were systematically compared with the SEL competencies outlined in the TCEM framework, enabling the identification of areas of alignment and divergence between policy design and classroom-level realities.

To enhance analytic transparency, the final coding structure and thematic map were exported from NVivo 14 and visualized in MAXQDA 2024. MAXQDA 2024 was used at this stage because it offers flexible graphical tools for representing hierarchical relationships between themes and subthemes. MAXQDA was used solely for the graphical representation of themes and subthemes and did not inform additional coding or analytic decisions. The resulting thematic map is presented in Figure 1. To further support transparency and replicability, the complete codebook, including code definitions and the final thematic framework, is openly accessible via the Open Science Framework (OSF): https://osf.io/qwkth/files/bucv3.

Throughout the analytic process, attention was given to both cross-site consistencies and regional variations, ensuring that context-specific or less dominant perspectives were not overshadowed by more prevalent patterns. Verbatim quotations are included to illustrate analytic interpretations and preserve participants' voices. All participants were anonymised using focus group and teacher identifiers (e.g., FG1-T1, FG3-T4). Due to the geographically dispersed participant pool and the group-based nature of the data collection, transcripts and analytic interpretations were not returned to participants for formal member checking. Instead, credibility was supported through collaborative coding, iterative discussions among the research team during analysis, and the use of verbatim quotations to ensure that interpretations remained closely grounded in participants' accounts.

### 3.4 Rigor in the research

We ensured methodological rigor through strategies aligned with *credibility, transferability, dependability, and confirmability* as articulated by Lincoln and Guba (1985), alongside Tracy's (2010) principles of *rich rigor and multivocality*. Rather than treating rigor as a procedural checklist, we approached it as an ongoing analytic commitment embedded across all stages of the research process.

We established credibility through prolonged and repeated engagement with the data. All transcripts were read multiple times by the research team, and analytic interpretations were discussed in regular peer debriefing meetings during coding and theme development. These discussions served to interrogate emerging interpretations, surface implicit assumptions, and refine analytic boundaries. In addition, we actively searched for negative cases and disconfirming evidence to avoid overgeneralisation and to strengthen interpretive depth (Shenton, 2004). The findings are illustrated with extended verbatim excerpts drawn from teachers across different regions, demonstrating clear grounding in the data.

We supported transferability by providing thick descriptions of the study context, participant characteristics, and analytic procedures. The use of maximum variation sampling—including teachers from diverse geographical regions, school contexts, and ranges of professional experience—enabled the exploration of SEL experiences across heterogeneous educational settings. These contextual details allow readers to assess the relevance and applicability of the findings to other settings.

We addressed dependability and confirmability through a systematic and transparent analytic process. Coding followed Saldaña's (2021) recommendations, beginning with data-near descriptive coding in the first cycle and progressing to pattern coding in the second cycle. The use of NVivo supported the organization, traceability, and auditability of the coding process, while analytic decisions, theme revisions, and reflexive memos were documented throughout analysis (Nowell et al., 2017). Raw data (audio recordings and transcripts) were securely stored, and all analytic steps were preserved to enable methodological transparency.

Consistent with the reflexive orientation of thematic analysis, the researchers adopted an explicitly reflexive stance, recognizing that interpretation is shaped by researchers' theoretical positions, disciplinary backgrounds, and prior engagements with SEL research. Rather than seeking neutrality, reflexivity functioned as a resource for critically examining how meanings were co-constructed during analysis.

Ethical approval was granted by the Osmangazi University Social and Human Sciences Human Research Ethics Committee (Decision No. 2025-14; 21 August 2025). All participants provided informed consent prior to participation (For consent forms, see OSF; https://osf.io/qwkth/files/fcvw7). Participation was voluntary, confidentiality was ensured through anonymisation procedures, and teachers retained the right to withdraw from the study at any time without consequence.

## 4 Results

**Figure 2:**
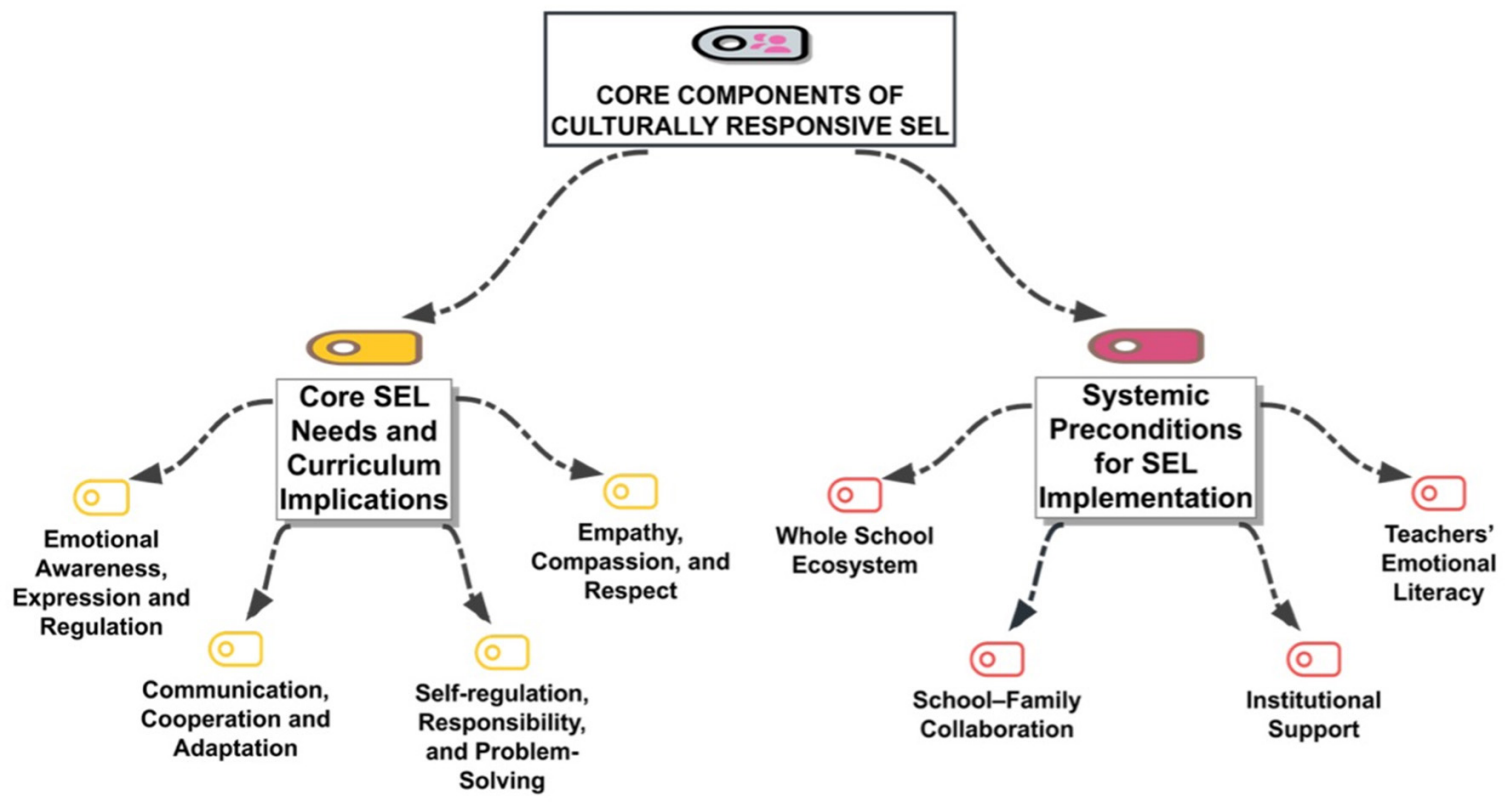
Core components of culturally responsive SEL in Türkiye.

The research findings reveal a coherent configuration of SEL needs while identifying the critical systemic preconditions necessary for their sustainable implementation. These themes are integrated into a conceptual framework of Culturally Responsive SEL (Figure 2), which maps the relationship between core curriculum implications and the broader institutional support ecosystem.

### 4.1 Core SEL needs and curriculum implications: a needs-to-design logic

When teachers were asked to identify the social and emotional competencies most urgently needed by their students, their responses revealed a clear and consistent pattern across regions and school contexts. Teachers described a closely linked set of unmet needs stemming from daily classroom situations, such as emotional outbursts, disputes among peers, communication breakdowns, and students' limited ability to regulate their emotions and engage with others.

#### 4.1.1 Emotional awareness, expression and regulation

##### 4.1.1.1 Observed need

Across focus groups, teachers consistently identified students' limited emotional awareness, expression, and regulation as a central social–emotional learning need. At the most basic level, many children were described as struggling to recognize and label their own emotional states. Teachers noted that students often relied on vague descriptors such as “good” or “bad,” or were unable to connect emotions to concrete lived experiences. As one teacher explained, children who cannot articulate their inner states remain emotionally inaccessible, “like closed boxes,” disconnected from their families, peers, and surroundings (FG7-T2).

Difficulties in emotional awareness were closely linked to limited emotional expression. Teachers reported that in many classrooms, only one student—or sometimes none—could clearly express emotions verbally. Emotional vocabulary itself was described as strikingly underdeveloped, not only among students but across educational contexts more broadly. One participant remarked that even adults would struggle to produce a wide range of emotion statements (FG10-T1; FG7-T3). Several teachers traced these challenges to early childhood contexts, observing that many students entered school without opportunities to name or discuss emotions within their families and were unable to respond even to simple prompts such as identifying what makes them happy or sad (FG5-T5).

Teachers consistently described emotional expression and emotional regulation as inseparable. When children were unable to recognize or verbalize emotions such as jealousy, anger, or frustration, these emotions were reported to surface through behavioral escalation in peer interactions. One teacher explicitly linked unrecognized jealousy to bullying behavior, emphasizing that regulation difficulties stemmed from unarticulated emotional experience rather than deliberate misconduct (FG10-T3). Importantly, teachers did not frame emotions as something to be eliminated. Instead, they stressed that the core educational challenge lay in helping children learn how to manage emotions constructively within appropriate boundaries. As one teacher stated, “emotions such as anger or love cannot be removed; what matters is learning how to regulate them” (FG10-T7).

Taken together, teachers' accounts portray emotional awareness, emotional expression, and emotional regulation as sequential and interdependent capacities. When children cannot recognize emotions, they struggle to express them; when emotions remain unexpressed, they are more likely to be enacted through dysregulated behavior. Teachers thus positioned emotional awareness and expression as foundational needs upon which communication, social participation, and responsible behavior depend (FG10-T3).

##### 4.1.1.2 Curricular implications

These accounts strongly suggest that SEL curricula must move beyond emotion naming to explicitly support emotional expression and regulation, including regulation strategies, coping mechanisms, and self-soothing practices. Teachers implicitly rejected assumptions that emotional competence develops naturally once emotions are labeled. Instead, they emphasized the need for structured, repeated opportunities to practice articulating emotions, managing frustration, calming down, and responding proportionally to emotional triggers.

Crucially, teachers' reflections revealed that emotion regulation cannot be effectively taught without teachers' own emotional awareness and reflexivity. Several participants described a professional learning trajectory in which they had to unlearn reactive or punitive responses and develop more empathic, reflective practices. One teacher candidly contrasted early-career tendencies to shout or demand compliance with later approaches grounded in empathy and perspective-taking (FG10). Teachers also noted that students closely observe how adults manage emotions in moments of stress, conflict, or error. When teachers model calmness, acknowledge mistakes, or apologize, children are said to internalize these behaviors as legitimate emotional responses.

#### 4.1.2 Communication, cooperation and adaptation

##### 4.1.2.1 Observed need

Across focus groups, communication was repeatedly described as the most fundamental skill, as one participant emphasized, “Communication is the most basic skill… if that improves, everything improves” (FG11-T3). Teachers reported that many students struggled to engage in meaningful interaction beyond familiar classroom contexts; even academically successful children were described as becoming withdrawn, silent, or avoidant when required to communicate with unfamiliar adults, peers or even school staff (FG7-T3). Teachers frequently linked communication difficulties to children's home environments, noting limited opportunities for dialogue and active listening. One teacher described how constant background television, crowded families, and the absence of conversational space at home resulted in children who “cannot talk, cannot express themselves” (FG6-T4).

Closely related to communication, cooperation emerged as another salient unmet need. Teachers described widespread difficulties in sustaining group work, sharing responsibility, and resolving disagreements without adult intervention. Students were reported to abandon collaborative tasks quickly, compete for dominance, or disengage entirely, requiring teachers to act as constant “referees” (FG3; FG11). One teacher noted that children “cannot sustain cooperation at all” and tend to resolve conflicts “through fighting rather than negotiation” (FG3-T13). These challenges were often attributed to family practices characterized by overprotection or “helicopter parenting,” which limited children's opportunities to practice teamwork and shared problem-solving (FG11-T7).

Teachers also highlighted adaptation to classroom norms and social rules as an unmet SEL need. Adaptation was not framed as compliance, but as awareness of context and consideration for others. One teacher described this as “being aware that one is in a classroom and not disrupting the shared learning environment” (FG1-T2), while another emphasized the importance of adjusting behavior to school culture and collective expectations (FG3-T2).

##### 4.1.2.2 Curricular implications

Teachers' accounts suggest that SEL curricula should explicitly prioritize communication, cooperation, and social adaptation as teachable competencies rather than assuming their natural development through classroom exposure. Communication emerged as a foundational skill requiring systematic and repeated practice. Teachers implicitly pointed to pedagogical approaches such as drama, role-play, structured dialogue, and cross-context speaking opportunities as effective tools for normalizing self-expression and reducing communicative inhibition.

Similarly, cooperation should be intentionally scaffolded through developmentally appropriate group work with clear roles, shared goals, and guided interaction. Teachers' experiences indicate that, in the absence of explicit curricular support, group activities often deteriorate into conflict or require constant adult mediation. Social and classroom adaptation—including turn-taking, respect for shared norms, and participation in collective routines—should therefore be embedded across subject areas and everyday classroom practices, ensuring that SEL is enacted consistently rather than treated as an add-on.

At the same time, teachers emphasized that communicative development is highly contingent on teachers' interactional stance. Children's willingness to speak was described as sensitive to whether teachers adopt a curious and non-judgmental language (“what might be behind this behavior?”) rather than blaming or dismissive responses that silence student voice (FG2). This underscores that supporting students' communication also requires strengthening teachers' own emotional vocabulary and reflective communicative practices, positioning self-expression as an adult as well as a child learning demand (FG7).

#### 4.1.3 Self-regulation, responsibility, and problem-solving

##### 4.1.3.1 Observed need

Across focus groups, teachers consistently described students' difficulties with self-regulation as a foundational social–emotional learning need that shaped multiple dimensions of school functioning. Self-regulation was defined as the capacity to plan, sustain attention, tolerate frustration, and complete tasks without continuous adult prompting. Teachers frequently referred to students who were unable to persist, wait their turn, or manage everyday routines independently, particularly in play and classroom transitions. Difficulties with patience and impulse control were often cited as triggers for conflict, especially during games, where minor frustrations quickly escalated (FG5-T3).

Building on this core difficulty, responsibility and responsible decision-making emerged as the social and moral extension of weak self-regulation. Responsibility was framed not merely as task completion, but as the ability to act independently, make choices, and accept the consequences of one's actions. Teachers repeatedly contrasted students who could prepare their own materials, follow routines, and persist despite mistakes with those who required constant reassurance and supervision. Several participants explicitly linked this dependence to individuation, arguing that students struggled to take responsibility because they were accustomed to acting under continuous adult direction rather than personal choice (FG7-T3). One teacher described students who needed “someone over them like a shepherd,” emphasizing that such children shaped their behavior around adult expectations rather than internal standards (FG11).

Teachers overwhelmingly attributed these patterns to family practices characterized by excessive monitoring and intervention. Examples included parents packing school bags, completing homework, or seeking immediate confirmation for minimal tasks. One teacher illustrated this dynamic by describing students who repeatedly asked for validation after writing a single word on the board, mirroring home environments where every action was instantly corrected or approved (FG3-T14). Across accounts, teachers emphasized that while such practices were often well-intentioned, they limited children's opportunities to practice self-regulation and responsibility, reinforcing dependence on external authority.

Within this context, problem-solving and coping were described as the situational enactment of both self-regulation and responsibility. Teachers framed problem-solving not in academic terms, but as the ability to confront difficulties, seek solutions, and recover from setbacks without emotional collapse. Successful students were described as those who believed that “there is a solution to every problem,” who did not give up when faced with challenges, and who knew when and how to seek help appropriately (FG1-T2; FG7-T3). Several teachers emphasized that children who could cope with frustration, adapt to new situations, and persist through difficulty were better equipped not only for school demands but for life more broadly, describing these capacities as what ultimately “keeps a child standing” (FG5-T3). Taken together, teachers' accounts suggest that self-regulation, responsibility, and problem-solving form an interdependent cluster of capacities underpinning student autonomy.

##### 4.1.3.2 Curricular implications

Teachers' accounts indicate that SEL curricula should position self-regulation as an explicit instructional focus. Executive-function skills such as planning, persistence, impulse control, and frustration tolerance need to be deliberately taught and practiced through everyday curricular routines (e.g., task initiation, transitions, play, and homework planning), with intentional fading of adult support.

Responsibility and responsible decision-making should be treated as curricular outcomes of supported autonomy. This requires designing learning tasks that offer real choices, require follow-through, and allow students to experience consequences in low-risk contexts, rather than over-scaffolded or adult-managed routines.

Problem-solving and coping should be embedded as the situational enactment of self-regulation and responsibility across subjects. Curricula should normalize challenge by providing repeated opportunities to face difficulty, consider alternatives, seek help appropriately, and recover from setbacks.

#### 4.1.4 Empathy, compassion, and respect

##### 4.1.4.1 Observed need

Across focus groups, teachers consistently identified empathy, together with compassion and respect, as one of the most urgent and insufficiently developed social–emotional needs among students. Teachers repeatedly described peer cultures characterized by cruelty, impatience, and disregard for others, emphasizing that many children failed to recognize how their actions affected classmates. As one group stated bluntly, “they are very cruel to each other… they hurt one another deeply” (FG3). This lack of empathy was frequently illustrated through everyday classroom incidents, such as laughing when a peer cried, refusing to share, or escalating conflicts during games. Several teachers noted that children often acted without any awareness of others' feelings: “when a child does something, they cannot understand at all what the other person is feeling” (FG7-T4). In more extreme cases, teachers described a troubling absence of compassion toward vulnerable beings, reinforcing concerns about children's moral sensitivity (FG1-T5).

Alongside empathy, respect emerged as a recurrent and closely intertwined concern. Teachers across regions explicitly stated that students lacked basic respect toward peers, teachers, and shared classroom norms, describing respect as “the most fundamental” social value needed in school life (FG9-T15). Everyday examples—such as interrupting others, refusing to wait one's turn, or ignoring peers' needs—were framed as manifestations of respect deficits rather than mere behavioral issues. As one teacher noted, “almost all of our problems stem from disrespect toward one another” (FG9-T15).

Teachers consistently framed empathy, compassion, and respect as inseparable, arguing that the absence of one disrupted the entire social fabric of the classroom. Several participants emphasized that compassion and conscience were among the last-developing qualities in children, particularly in contexts marked by indulgent or overprotective family practices (FG9-T21). Children raised as unquestioned “princes or princesses,” teachers argued, struggled to recognize others' perspectives and limits (FG5-T3).

Importantly, teachers did not treat these capacities as optional traits or personality differences. Instead, empathy, compassion, and respect were positioned as moral foundations of social–emotional learning, shaping classroom climate, peer cooperation, and even academic engagement. As one teacher stressed, “if empathy and tolerance are there, I don't even care if the child can say two times two is four” (FG10-T2). In this sense, teachers framed moral–relational competencies not as outcomes of success, but as the conditions that make meaningful participation in school life possible.

##### 4.1.4.2 Curricular implications

Teachers' accounts suggest that empathy, compassion, and respect must be treated as explicitly teachable SEL competencies, not assumed by-products of social interaction or value statements. SEL curricula should therefore embed guided perspective-taking, reflection on everyday peer incidents, and structured cooperative activities that require sharing, listening, and attending to others' needs. Respect should be operationalised through clear, practiced classroom norms (e.g., turn-taking, boundaries, care for others) that are reinforced across subjects and routines. Finally, teachers' emphasis on classroom climate indicates that these competencies are most effectively developed when teachers model empathic language and responses, positioning empathy and respect as organizing principles of daily classroom practice rather than isolated SEL topics.

### 4.2 Systemic preconditions for SEL implementation

Beyond individual competencies, teachers consistently emphasized that the effectiveness of social and emotional learning program depends on a set of systemic and structural conditions extending beyond the classroom. Across regions, SEL was not conceptualized merely as a student-level skills framework, but as a whole-school and whole-ecosystem endeavor requiring alignment among families, educators, and institutional resources.

Family involvement emerged as the most critical systemic prerequisite. Teachers repeatedly noted that students often returned to school environments that contradicted the values and practices promoted through SEL, such as empathy, emotional regulation, or respectful communication (FG1; FG10). In such cases, classroom-based SEL efforts were described as fragile and easily undermined. As one teacher stated, “without family awareness, SEL does not work” (FG7), while others argued that teachers' efforts “collapse” in the absence of parental alignment. Consequently, many participants called for systematic and mandatory parent education, rather than voluntary or *ad hoc* involvement.

Teachers further emphasized that families themselves often require emotional literacy support. In regions such as FG2, participants highlighted cases in which children's behavioral and emotional difficulties were rooted in trauma histories, migration experiences, or family stress, necessitating coordinated school–family collaboration. Some teachers described practical strategies, such as modeling SEL practices during parent meetings (FG4), and reported noticeable improvements in children's behavior when parents understood and validated SEL approaches.

Beyond family engagement, institutional support structures were also seen as indispensable. Teachers stressed the need for access to guidance counselors and psychological support services, particularly in contexts marked by displacement, socio-economic hardship, or emotional vulnerability. Similarly, play- and drama-based pedagogies were repeatedly highlighted as essential modalities for SEL, allowing children to experience, enact, and regulate emotions in embodied and relational ways. The availability of adequate physical space—such as multipurpose rooms, playgrounds, or areas suitable for drama and group activities—was likewise identified as a prerequisite for sustained SEL implementation.

Importantly, teachers also positioned themselves as central agents within this ecosystem. Several accounts emphasized that SEL is learned not only through explicit instruction, but through the daily observation of adult behavior. Teachers highlighted the importance of consistency, emotional self-regulation, and modeling respect, empathy, and responsibility in everyday interactions. From this perspective, teachers' own social–emotional competencies were framed not as individual attributes, but as systemic conditions that shape classroom climate and legitimize SEL practices through lived example.

## 5 Discussion

### 5.1 What SEL is needed now? Teachers' priority competencies

Social and emotional learning is inherently culturally situated rather than culturally neutral. Norms governing emotional expression, communication, cooperation, and self-regulation are shaped by local moral expectations, relational practices, and institutional routines, and therefore vary across cultural, social, and educational contexts (Jones et al., 2021). What counts as appropriate emotional behavior or effective social participation is not universally fixed, but locally negotiated. From this perspective, SEL is best understood not as a standardized set of decontextualised skills, but as a culturally responsive practice that reflects the everyday realities, interactional norms, and value systems of the communities in which it is enacted (Jukes et al., 2018). Guided by this premise, the present study examines teachers' situated understandings of students' social–emotional learning (SEL) needs in Turkish primary school contexts.

Across teachers' accounts, priorities converged around four interrelated clusters of competencies consistently described as critical for students' participation in everyday school life. Emotional awareness, expression, and regulation were foregrounded as foundational capacities, reflecting concerns about children's ability to recognize inner states and manage emotional reactions in socially appropriate ways. Closely connected to this, communication, cooperation, and adaptation to classroom norms were emphasized as essential for functioning within collective learning environments, where speaking, listening, negotiating, and complying with shared rules were frequently associated with being a “good” student. Teachers also highlighted self-regulation, responsibility, and problem-solving as pressing needs, particularly in relation to students' dependence on adults and difficulties sustaining effort or coping with challenges independently. Finally, empathy, compassion, and respect were framed as moral–relational orientations shaping how children treat peers, manage differences, and contribute to a cohesive classroom climate. Taken together, this clustering indicates that teachers conceptualize SEL not as a collection of discrete skills, but as a set of interdependent capacities that enable children to regulate themselves and relate to others within shared social spaces. The simultaneous emphasis on self-related and relational competencies reflects an underlying concern with balancing individual self-management and relational harmony in everyday classroom functioning.

Interpreting these priorities through the lens of Türkiye's socio-cultural profile helps clarify why competencies related to emotional regulation, communication, responsibility, and empathy were foregrounded. Although the individualism–collectivism distinction represents a broad and necessarily simplified way of characterizing cultural orientations (Kagitçibaşi and Berry, 1989), it remains a useful heuristic for understanding how social expectations shape relational behavior and self-regulation in educational contexts. Research on Türkiye's cultural positioning within this continuum has yielded mixed findings. While some studies describe Türkiye as predominantly collectivist, emphasizing interdependence and group harmony (Hofstede, 2011; Minkov and Kaasa, 2022; Suh et al., 1998), others point to a more complex configuration in which collectivist orientations coexist with individualistic tendencies such as personal agency, social sensitivity, and active involvement in others' lives (Göregenli, 1997; Imamoglu and Gültekin-Yasak, 1993; Uskul, 1998). Cross-national comparisons further situate Türkiye closer to the collectivist end of the spectrum when contrasted with highly individualistic contexts such as the United States (Hofstede Insights, 2024).

Within Türkiye's culturally ambivalent context, the present findings become more intelligible. Teachers' simultaneous emphasis on emotional awareness, self-regulation, responsibility, communication, and empathy suggests an orientation that values individual self-management insofar as it enables harmonious participation in collective settings. This pattern aligns closely with Kagitçibaşi's (1996) conceptualization of Turkish culture as autonomous–relational, in which emotional interdependence coexists with expectations for personal responsibility and autonomy. From this perspective, teachers' concerns about students' dependence on adults, difficulties with self-regulation, and challenges in cooperative interaction can be understood as tensions arising from navigating these dual cultural expectations in contemporary classrooms.

Rather than constituting a separate explanatory layer, generational characteristics may render these underlying cultural tensions more visible in everyday school life. Research on Generation Z in Türkiye describes young people as socially connected and expressive, yet simultaneously embedded within paternalistic and hierarchical relational frameworks that privilege indirect communication and asymmetrical authority relations (Nas and Dogan, 2020; Çil and Çevik, 2024; Hofstede Insights, 2024). Within such contexts, teachers' concerns regarding emotional dysregulation, reliance on adult intervention, and difficulties sustaining cooperative interaction may be understood not merely as behavioral deficits but as manifestations of a generationally inflected tension between relational connectedness and constrained autonomy—a tension that is culturally structured rather than individually determined.

A complementary developmental perspective emerges from research on emotion socialization in Turkish families (Kiliç et al., 2026). This work suggests that many Turkish children enter school having been socialized into emotional norms that prioritize restraint and context-sensitive expression over explicit verbal articulation of inner states. From this perspective, teachers' emphasis on emotional awareness, expression, and regulation may partly reflect a perceived discontinuity between the emotion repertoires cultivated in family contexts and the communicative expectations embedded in classroom participation. Such a home–school disjuncture underscores the importance of culturally situated approaches to SEL that recognize the developmental pathways through which emotional competencies are acquired, rather than assuming expressive verbal articulation as a universal baseline.

Evidence from person-centered research with Turkish adolescents further echoes this pattern. Using latent profile analysis, Agirkan and Haspolat (2025) identified three empirically distinct SEL profiles—SEL-Risk, Prosocial-Low Intrapersonal, and SEL-Competent. Particularly instructive is the Prosocial-Low Intrapersonal profile, characterized by relatively strong social awareness alongside markedly weaker intrapersonal competencies such as self-management, self-awareness, and responsible decision-making. This asymmetric configuration mirrors the relational pattern described by teachers in the present study: students who appear socially attuned to others yet struggle with independent self-regulation within shared classroom contexts. Although the present findings are grounded in teachers' situated perceptions rather than student-level measurement, the convergence between these qualitative insights and emerging quantitative evidence suggests that such patterns may reflect broader developmental dynamics within Turkish school contexts.

Beyond these cultural and developmental interpretations, the configuration of SEL priorities identified in the present study also both converges with and extends existing national research conducted in Türkiye. Bacanli et al. (2022) provided one of the earliest needs-based examinations of SEL from Turkish educators' perspectives, identifying communication, empathy, self-awareness, self-regulation, and relationship-building as foundational competencies. While that study made an important contribution by articulating a preliminary conceptual model of SEL within the Turkish educational context, its reliance on survey responses and a sample drawn primarily from three metropolitan cities limited its ability to capture regional diversity and teachers' situated interpretations of SEL as enacted in everyday classroom life. By contrast, the present multi-site qualitative study, drawing on focus groups with teachers across 26 cities, substantially broadens this evidentiary base by illuminating how SEL priorities are interpreted, negotiated, and enacted across diverse socio-cultural and educational contexts.

Cross-cultural evidence further situates these findings within a broader comparative landscape. Drawing on educators' perspectives across several Global North countries, including Türkiye, Kourmousi et al. (2025) identified a wide range of SEL competencies expected of students, organized into interpersonal skills, social awareness skills, decision-making skills, self-awareness skills, self-regulation skills, and self-efficacy and self-esteem skills, with interpersonal skills and social awareness emerging as the most frequently emphasized domains. This configuration closely aligns with the present study's identification of communication, cooperation, empathy, and regulation as central SEL needs in Turkish classrooms. However, whereas the survey-based, cross-national design of Kourmousi et al. (2025) highlights convergence in which competencies are valued across contexts, the present study adds analytic depth by clarifying how these competencies are interpreted, prioritized, and enacted within everyday classroom interactions.

In particular, the present findings extend Kourmousi et al.'s (2025) work in two important ways. First, while self-regulation and decision-making in their study are discussed largely as individual competencies associated with effectiveness and future readiness, teachers in the present study framed self-regulation and responsibility primarily as capacities enabling behavioral stability, independent functioning, and appropriate participation within shared classroom contexts. In this sense, self-management was valued less as an individual achievement resource and more as a prerequisite for sustaining collective classroom life. Second, the prominence of self-awareness identified by Kourmousi et al. (2025) can be productively reinterpreted through the lens of emotional awareness, expression, and regulation foregrounded in the present study. Whereas self-awareness in the broader SEL literature is often linked to recognizingv strengths and limitations for academic or career-related decision-making within exam-centered systems, teachers in the present study described awareness primarily in emotional and relational terms—recognizing feelings, articulating internal states, and regulating emotional responses in interaction with others.

Our practice-oriented interpretation is further reinforced by international research focusing specifically on empathy. Studies by Kounenou et al. (2025) and Scoda and Park (2025) conceptualize empathy as a multifaceted construct encompassing perspective-taking, emotional attunement, respect for diversity, and communicative competence across diverse cultural contexts. While these studies demonstrate strong cross-national consensus regarding the importance of empathy, the present study contributes a culturally grounded reinterpretation by clarifying how empathy becomes visible in classroom life. Teachers described empathy as meaningful only when enacted through compassionate behavior, restraint, fairness, and consideration for others in everyday interactions. In this sense, empathy was experienced not as an internal disposition or abstract emotional skill, but as an enacted pedagogical outcome emerging through communication, emotional regulation, and relational conduct. Empathy, from teachers' perspectives, becomes evident only when emotional understanding is translated into appropriate action—suggesting that SEL curricula focusing primarily on emotional insight without behavioral enactment may remain misaligned with teachers' lived criteria for empathic competence.

Taken together, these comparisons indicate that while existing national and cross-cultural studies successfully identify which SEL competencies matter, the present study advances the field by clarifying how these competencies are lived, prioritized, and enacted in context. By integrating regional diversity, cultural orientations, and teachers' situated meaning-making, this study reframes SEL in Türkiye as a relational and practice-based construct—one that aligns with international frameworks in content, yet diverges in its emphasis on collective functioning, moral responsibility, and everyday enactment.

### 5.2 From skills to systems: why teachers conceptualize SEL as an ecosystem

Teachers' accounts indicate that social and emotional learning is not conceptualized as a discrete set of teachable skills confined to individual students or isolated classroom activities. Instead, SEL is framed as an ecosystemic process that emerges through the interaction of students, teachers, families, school structures, and broader social conditions. In this sense, SEL was described not as something that can be “delivered” through lessons alone, but as something that is sustained or undermined by the degree of alignment across environments in which children participate.

Classroom-based SEL efforts were repeatedly characterized as fragile when children returned to home or community contexts that did not reinforce, or actively contradicted, SEL values such as emotional regulation, empathy, and respectful communication. This emphasis aligns with ecological theory's focus on the mesosystem as a critical site of developmental influence, where consistency across settings amplifies—or erodes—learning and behavior (Bronfenbrenner, 1977). Empirical evidence similarly demonstrates that SEL interventions incorporating family–school components yield stronger gains in children's prosocial behavior, peer relationships, and mental health than school-only approaches (Agirkan et al., 2023; Garbacz et al., 2025). Notably, even low-intensity but consistent forms of home–school communication have been shown to produce meaningful effects on social–emotional competence and wellbeing (Sheridan et al., 2019), underscoring the leverage of relational continuity across contexts.

Within this broader ecosystem, teachers positioned institutional support as a collective rather than individual responsibility. Access to guidance services, psychological support, adequate physical spaces, and coherent school-level coordination were consistently described as prerequisites for effective SEL. These observations resonate with research on collective efficacy, which conceptualizes improvement as dependent on shared beliefs about a group's capacity to act in coordinated ways to achieve desired outcomes (Bandura, 2000; Goddard et al., 2000). From this perspective, SEL success cannot be sustained through isolated teacher effort, but requires organizational structures that distribute responsibility across the school community.

Crucially, teachers' ecosystemic framing also foregrounds the role of educator SEL as a central enabling condition within this system. Educator SEL encompasses the dispositions, skills, and reflective capacities that allow teachers to create emotionally supportive environments, integrate SEL into academic content, and model competencies through everyday interaction (Jones and Kahn, 2017; Wanless and Domitrovich, 2015). Teachers' accounts implicitly positioned SEL as something students learn not only through instruction, but through observation—by watching how adults regulate emotions, navigate conflict, and relate to others under pressure. Research supports this interpretation. Effective educators make SEL visible by narrating their own use of social–emotional skills, including the ongoing unlearning of counterproductive mindsets and reactive practices (Jennings and Alamos, 2024). Intentional modeling of emotional regulation, empathy, and prosocial behavior in daily exchanges has been consistently associated with improvements in classroom climate (Alemdar et al., accepted; Özen-Uyar et al., 2025) as well as gains in students' social–emotional and academic outcomes (Agirkan et al., 2023; Shi and Cheung, 2024). In this sense, educator SEL operates not as an add-on to curriculum, but as the relational infrastructure through which SEL becomes credible, coherent, and learnable.

### 5.3 Alignment and tensions with TCEM: from classroom reality to national SEL framework

Teachers' accounts in the present study reveal a coherent configuration of social–emotional learning needs centered on emotional awareness, expression, and regulation; communication, cooperation, and adaptation; self-regulation, responsibility, and problem-solving; and empathy-based relational conduct. At a conceptual level, this configuration aligns closely with the Türkiye Century Education Model (TCEM), which organizes SEL into self-skills, social life skills, and compound skills. Teachers' emphasis on recognizing emotions, managing reactions, and sustaining effort independently corresponds with TCEM's self-skills domain, while their focus on communication, cooperation, and adaptation aligns with social life skills. Similarly, teachers' concerns regarding responsibility, coping with challenges, and decision-making resonate with TCEM's compound skills, including adaptability and responsible decision-making. This convergence reflects broader SEL scholarship that conceptualizes social–emotional competence as an integrated system of intrapersonal regulation, interpersonal engagement, and responsible action [Collaborative for Academic, Social, and Emotional Learning (CASEL), 2025; World Health Organization, 2003].

Viewed through this lens, teachers' accounts meaningfully extend and partially reframe TCEM's architecture. While TCEM distributes identity, values, and dispositions across parallel components of the model (e.g., the Virtue–Value–Action framework and dispositions), teachers' pedagogical meaning-making suggests that identity, emotional regulation, and relational competence are deeply intertwined in practice. Teachers did not distinguish between emotional skills, moral orientation, and relational behavior; instead, they described SEL needs as emerging at the intersection of these domains, where emotional awareness informs regulation, regulation enables communication, and communication sustains empathy and respect. This pattern highlights a potential misalignment between TCEM's compartmentalized structure and teachers' holistic enactment of SEL in classrooms.

Teachers' accounts also sharpen a practical–operational tension inherent in centralized, textbook-driven systems. Although TCEM delineates which social–emotional skills should develop, it provides limited guidance on how these skills are to be made visible, teachable, and sustainable within everyday classroom routines. When SEL is framed primarily as a transversal aspiration without explicit subject-level integration, enactment becomes uneven and highly dependent on individual teacher initiative. This concern aligns with implementation research demonstrating that effective SEL requires not only clearly defined competencies, but pedagogical approaches that are sequenced, active, focused, and explicit—the SAFE practices identified as central to successful SEL programming (Durlak et al., 2011). Such sequencing is particularly critical given evidence that social–emotional competencies build on one another, with foundational skills such as emotional awareness and regulation serving as prerequisites for more complex capacities like conflict resolution and responsible decision-making (Jones et al., 2021; Bailey and Jones, 2019). From teachers' perspectives, competencies such as emotion regulation, empathy, and responsible decision-making therefore do not reliably “emerge organically” through participation or values discourse alone, but require developmentally ordered and pedagogically supported instruction.

Finally, teachers' accounts point to the importance of pedagogical support structures that extend beyond curriculum design. Teachers repeatedly described SEL as fragile when responsibility rested solely on individual effort, indicating a need for sustained professional learning rather than isolated materials. Evidence from large-scale SEL programmes similarly shows that ongoing coaching, lesson modeling, and collaborative reflection are critical for supporting teachers' enactment of SEL in practice (Jones et al., 2010). Taken together, these findings suggest that while TCEM offers a conceptually aligned framework, its impact depends on the extent to which SEL is supported through explicit pedagogy and continuous teacher development.

## 6 Policy and curriculum implications

The findings suggest that culturally responsive SEL policy and curriculum in Türkiye would benefit from moving beyond broad competency frameworks toward a more practice-oriented and relational design. Teachers experienced SEL primarily as capacities enacted in everyday classroom life—through recognizing and naming emotions, regulating reactions, negotiating interaction, and repairing relationships—rather than as abstract skills addressed separately from instruction. These competencies were understood as developing through situated and embodied learning, in which modeling, guided practice, and real-time feedback within authentic routines played a central role. Effective implementation, therefore, depends not only on clearly articulated SEL domains, but also on pedagogical approaches that are sequenced, active, focused, and explicit, alongside sustained teacher professional development that strengthens teachers' own social–emotional competencies, pedagogical strategies and reflective practices. In highly centralized and textbook-driven systems, the absence of such pedagogical support risks rendering SEL uneven and fragile, particularly when academic priorities dominate classroom time. Teachers' accounts further underscore the importance of approaching SEL as a whole-school endeavor, where coherence between classroom practices, school culture, and family expectations supports shared responsibility and relational continuity. Within this context, conceptualizing SEL as a set of transferable core practices or “kernels” may offer a pragmatic policy response, enabling social–emotional learning to remain visible and actionable within everyday teaching routines rather than being overshadowed by academic content.

## Data Availability

The datasets presented in this article are not readily available because of ethical restrictions and the sensitive nature of qualitative interview data that could potentially enable the identification of participants. Anonymised codebooks is openly accessible via the Open Science Framework (OSF) at: https://osf.io/qwkth/files/bucv3. Requests to access the datasets should be directed to melek.alemdar@manchester.ac.uk.

## References

[B1] Agirkan M. Ergene T. (2022). What does the social and emotional learning interventions (SEL) tell us? A meta-analysis. Rev. Psicodidact. 27, 97–108. doi: 10.1016/j.psicod.2022.01.001

[B2] Agirkan M. Haspolat N. K. (2025). Profiles of social and emotional learning skills in adolescents: a latent profile analysis. Camb. J. Educ. 55, 333–355. doi: 10.1080/0305764X.2025.2502944

[B3] Agirkan M. Keklik I. Ergene T. (2023). The effects of student and school characteristics on SEL skills of adolescents: a hierarchical linear modelling. Psychol. Sch. 60, 4758–4781. doi: 10.1002/pits.23031

[B4] Albright T. N. Marsh J. A. Kennedy K. E. (2019). Social-emotional learning practices: insights from outlier schools. J. Res. Innov. Teach. Learn. 12, 35–52. doi: 10.1108/JRIT-02-2019-0020

[B5] Alemdar M. (2025). Mapping the evolution of social and emotional learning research in primary education contexts: a bibliometric and thematic analysis. J. Intell. 13:123. doi: 10.3390/jintelligence13090123 41003263 PMC12471157

[B6] Alemdar M. Qualter P. Wigelsworth M. O'Brien A. Hamilton S. Humphrey N. (accepted). Using core components in process evaluation: Passport skills for Life. PLoS ONE. 10.1371/journal.pone.0346416 PMC13037957 41915673

[B7] Alsaigh R. Coyne I. (2021). Doing a hermeneutic phenomenology research underpinned by Gadamer's philosophy: a framework to facilitate data analysis. Int. J. Qual. Methods 20, 1–10. doi: 10.1177/16094069211047820

[B8] Aşkar P. Altun A. (2023). K-12 beceriler çerçevesi: Türkiye bütüncül modeli üzerine bir çalişma [K-12 skills framework: a study on the Türkiye integrated model]. Milli Egitim Derg. 52, 925–940. Turkish. doi: 10.37669/milliegitim.1308740

[B9] Bacanli F. Ozdemir N. K. Ferrari L. Park C. M. Solberg V. S. H. (2022). Social emotional learning and career development from educators' perspectives grounded on the Turkish context. J. Career Dev. 49, 1402–1418. doi: 10.1177/08948453211050085

[B10] Bailey R. Jones S. M. (2019). An integrated model of regulation for applied settings. Clin. Child Fam. Psychol. Rev. 22, 2–23. doi: 10.1007/s10567-019-00288-y 30783912

[B11] Bandura A. (2000). Exercise of human agency through collective efficacy. Curr. Dir. Psychol. Sci. 9, 75–78. doi: 10.1111/1467-8721.00064

[B12] Biesta G. (2007). Bridging the gap between educational research and educational practice: the need for critical distance. Educ. Res. Eval. 13, 295–301. doi: 10.1080/13803610701640227

[B13] Blyth D. A. Jones S. Borowski T. (2018). SEL Frameworks–What Are They and Why Are They Important. Measuring SEL Data Practice 1, 1–9. Available online at: https://casel.s3.us-east-2.amazonaws.com/CASEL-Resources-Frameworks-What-Why.pdf (Accessed May 1, 2025).

[B14] Braun V. Clarke V. (2019). Reflecting on reflexive thematic analysis. Qual. Res. Sport Exerc. Health 11, 589–597. doi: 10.1080/2159676X.2019.1628806

[B15] Bronfenbrenner U. (1977). Toward an experimental ecology of human development. Am. Psychol. 32, 513–531. doi: 10.1037/0003-066X.32.7.513

[B16] Brush K. E. Jones S. M. Bailey R. Nelson B. Raisch N. Meland E. . (2022). “Social and emotional learning: from conceptualization to practical application in a global context,” in Life Skills Education for Youth: Critical Perspectives, eds. J. DeJaeghere, and E. Murphy-Graham (Berlin: Springer), 43–71. doi: 10.1007/978-3-030-85214-6_3

[B17] Chen L. Yu G. (2022). The impact of social-emotional learning: a meta-analysis in China. Front. Psychol. 13:1040522. doi: 10.3389/fpsyg.2022.1040522 36312196 PMC9597250

[B18] Chernyshenko O. S. Kankaraš M. Drasgow F. (2018). Social and Emotional Skills for Student Success and Well-Being: Conceptual Framework for the OECD Study on Social and Emotional Skills (OECD Educ. Work. Pap. 173). Paris: OECD Publishing.

[B19] Çil U. Çevik O. (2024). A novel operationalization approach in generation phenomenon: Insights and implications from Turkey's generational dynamics. J. Econ. Cult. Soc. 69, 133–148. doi: 10.26650/JECS2023-1296028

[B20] Cipriano C. Strambler M. J. Naples L. H. Ha C. Kirk M. Wood M. . (2023). The state of evidence for social and emotional learning: a contemporary meta-analysis of universal school-based SEL interventions. Child Dev. 94, 1181–1204. doi: 10.1111/cdev.13968 37448158

[B21] Collaborative for Academic Social, and Emotional Learning (CASEL). (2025). What is the CASEL Framework? Available online at: https://casel.org/fundamentals-of-sel/what-is-the-casel-framework/ (Accessed May 1, 2025).

[B22] Creswell J. W. Poth C. N. (2018). Qualitative Inquiry and Research Design: Choosing Among Five Approaches, 4th Edn. Thousand Oaks, CA: SAGE.

[B23] Demkowicz O. O'Brien A. Hamilton S. Burke L. Alemdar M. Mason C. . (2026). Child and school staff perceptions and experiences of universal social and emotional learning curricula in context: a qualitative case study registered report examining “Passport Skills for Life”. Br. J. Educ. Psychol. doi: 10.1111/bjep.70065. [Epub ahead of print]. 41772807 PMC13440978

[B24] Downes P. Cefai C. (2016). How to Prevent and Tackle Bullying and School Violence: Evidence and Practices for Strategies for Inclusive and Safe Schools (NESET II Report). Luxembourg: Publications Office of the European Union. Available online at: https://data.europa.eu/doi/10.2766/0799 (Accessed May 1, 2025).

[B25] Dray J. Bowman J. Campbell E. Freund M. Wolfenden L. Hodder R. K. . (2017). Systematic review of universal resilience-focused interventions targeting child and adolescent mental health in the school setting. J. Am. Acad. Child Adolesc. Psychiatry 56, 813–824. doi: 10.1016/j.jaac.2017.07.780 28942803

[B26] Durlak J. A. Weissberg R. P. Dymnicki A. B. Taylor R. D. Schellinger K. B. (2011). The impact of enhancing students' social and emotional learning: a meta-analysis of school-based universal interventions. Child Dev. 82, 405–432. doi: 10.1111/j.1467-8624.2010.01564.x 21291449

[B27] Dusenbury L. Weissberg R. P. (2018). Emerging Insights from States' Efforts to Strengthen Social and Emotional Learning. Chicago, IL: Collaborative for Academic, Social, and Emotional Learning. Available online at: https://files.eric.ed.gov/fulltext/ED586404.pdf (Accessed May 1, 2025).

[B28] Elias M. J. Zins J. E. Weissberg R. P. Frey K. S. Greenberg M. T. Haynes N. M. . (1997). Promoting Social and Emotional Learning: Guidelines for Educators. Alexandria, VA: Association for Supervision and Curriculum Development.

[B29] Esen-Aygün H. (2017). Sosyal-duygusal ögrenme programlarinin sosyal duygusal ögrenme becerilerinin gelişimine, akademik başari ve sinif iklimi algisina etkisi [The effect of social emotional learning program on development of social emotional learning skills, academic achievement and classroom environment] (Unpublished doctoral dissertation). Çanakkale Onsekiz Mart University, Çanakkale, Türkiye. Turkish.

[B30] Frechette J. Bitzas V. Aubry M. Kilpatrick K. Lavoie-Tremblay M. (2020). Capturing lived experience: methodological considerations for interpretive phenomenological inquiry. Int. J. Qual. Methods 19, 1–12. doi: 10.1177/1609406920907254

[B31] Gadamer H.-G. (1960/1989). Truth and Method, 2nd Edn. Transl. by J. Weinsheimer, and D. G. Marshall. New York, NY: Continuum.

[B32] Garbacz S. A. Ash T. L. Santiago R. T. (2025). “Family–school–community partnerships to promote SEL,” in Handbook of Social and Emotional Learning, 2nd Edn, eds. J. A. Durlak, C. E. Domitrovich, and J. L. Mahoney (New York, NY: The Guilford Press), 115–128.

[B33] Gay G. (2021). “Culturally responsive teaching: ideas, actions, and effects,” in Handbook of Urban Education, eds. H. R. Milner, and K. Lomotey (London: Routledge), 212–233. doi: 10.4324/9780429331435-16

[B34] Giorgi A. Giorgi B. (2003). “Phenomenology,” in Qualitative Psychology: A Practical Guide to Research Methods, ed. J. A. Smith (Thousand Oaks, CA: SAGE), 25–50.

[B35] Goddard R. D. Hoy W. K. Hoy A. W. (2000). Collective teacher efficacy: its meaning, measure, and impact on student achievement. Am. Educ. Res. J. 37, 479–507. doi: 10.3102/00028312037002479

[B36] Goodman A. Joshi H. Nasim B. Tyler C. (2015). Social and Emotional Skills in Childhood and Their Long-Term Effects on Adult Life. London: Institute of Education. Available online at: https://www.eif.org.uk/report/social-and-emotional-skills-in-childhood-and-their-long-term-effects-on-adult-life (Accessed November 1, 2025).

[B37] Göregenli M. (1997). Individualist-collectivist tendencies in a Turkish sample. J. Cross Cult. Psychol. 28, 787–794. doi: 10.1177/0022022197286009

[B38] Graves S. L. Herndon-Sobalvarro A. Nichols K. Aston C. Ryan A. Blefari A. . (2017). Examining the effectiveness of a culturally adapted social-emotional intervention for African American males in an urban setting. Sch. Psychol. Q. 32, 62–74. doi: 10.1037/spq0000145 27124505

[B39] Greenberg M. T. (2023). Evidence for Social and Emotional Learning in Schools. San Francisco, CA: Learning Policy Institute. doi: 10.54300/928.269

[B40] Ha C. McCarthy M. F. Strambler M. J. Cipriano C. (2025). Disentangling the effects of social and emotional learning programs on student academic achievement across grades 1–12: a systematic review and meta-analysis. Rev. Educ. Res. doi: 10.3102/00346543251367769

[B41] Heidegger M. (1927/1962). Being and Time. Transl. by J. Macquarrie, and E. Robinson. Manhattan, NY: Harper and Row.

[B42] Hofstede Insights (2024). Country Comparison: Turkey and the United States. Available online at: https://www.theculturefactor.com/country-comparison-tool?countries=turkey%2Cunited+states (Accessed January 14, 2026).

[B43] Hofstede G. (2011). Dimensionalizing cultures: the Hofstede model in context. Online Read. Psychol. Cult. 2:8. doi: 10.9707/2307-0919.1014

[B44] Humphrey N. (2013). Social and Emotional Learning: A Critical Appraisal. Thousand Oaks, CA: SAGE. doi: 10.4135/9781446288603

[B45] Humphrey N. Boehnke J. R. Santos J. Alemdar M. Panayiotou M. O'Brien A. . (2025). The effect of a universal, school-based social and emotional learning intervention (passport: skills for life) on internalizing symptoms and related outcomes during the transition from childhood to adolescence: a cluster-randomized controlled trial. J. Educ. Psychol. 117, 1095–1114. doi: 10.1037/edu0000963

[B46] Imamoglu E. O. Gültekin-Yasak Y. (1993). Önerilen dengelenmiş birey modeli işiginda üniversite ögrencilerinin sorunlari [University students' problems in light of the proposed balanced individual model]. Türk Psikol. Derg. 8, 27–41. Turkish.

[B47] Jennings P. A. Alamos P. (2024). “What is educator social and emotional learning (SEL)? Why is it important? How can it be promoted?” in Handbook of Social and Emotional Learning: Research and Practice, 2nd Edn, eds. J. A. Durlak, C. E. Domitrovich, and J. L. Mahoney (New York, NY: Guilford Press), 319–336.

[B48] Jones S. Bailey R. Meland E. Brush K. Nelson B. (2019). Tools for Selecting and Aligning International Frameworks for Social, Emotional, and Related Skills. The Taxonomy Project, Harvard Graduate School of Education. Available online at: https://echidnagiving.org/wp-content/uploads/2020/01/International-Frameworks-for-SEL.pdf (Accessed May 20, 2025).

[B49] Jones S. M. Bailey R. Brush K. Nelson B. Raisch N. Meland E. . (2021). Equity in social emotional learning programs: a content analysis of equitable practices in PreK-5 SEL programs. Front. Educ. 6:679467. doi: 10.3389/feduc.2021.679467

[B50] Jones S. M. Brown J. L. Hoglund W. L. G. Aber J. L. (2010). A school-randomized clinical trial of an integrated social–emotional learning and literacy intervention: impacts after 1 school year. J. Consult. Clin. Psychol. 78, 829–842. doi: 10.1037/a0021383 21114343

[B51] Jones S. M. Kahn J. (2017). The Evidence Base for How We Learn: Supporting Students' Social, Emotional, and Academic Development. Washington, DC: Aspen Institute. Available online at: https://www.aspeninstitute.org/wp-content/uploads/2017/09/SEAD-Research-Brief-9.12_updated-web.pdf (Accessed May 10, 2025).

[B52] Jukes M. Gabrieli P. Mgonda N. L. Nsolezi F. Jeremiah G. Tibenda J. . (2018). “Respect is an investment”: community perceptions of social and emotional competencies in early childhood from Mtwara, Tanzania. Glob. Educ. Rev. 5, 160–188.

[B53] Jukes M. C. H. (2019). “Contextualizing the goals of social and emotional learning curricula and materials,” in Educating for the Social, the Emotional and the Sustainable: Diverse Perspectives From Over 60 Contributors Addressing Global and National Challenges, eds. A. Smart, M. Sinclair, A. Benavot, J. Bernard, C. Chabbott, S. Garnett Russell, et al. (NISSEM), 182–197. Available online at: https://nissem.org/nissem-global-briefs/ngb-vol-i/ (Accessed May 20, 2025).

[B54] Kagitçibaşi C. (1996). The autonomous-relational self. Eur. Psychol. 1, 180–186. doi: 10.1027/1016-9040.1.3.180

[B55] Kagitçibaşi Ç. Berry J. W. (1989). Cross-cultural psychology: current research and trends. Annu. Rev. Psychol. 40, 493–532. doi: 10.1146/annurev.ps.40.020189.002425

[B56] Kaplan Z. Göl-Güven M. (2026). Teachers' SEL identity (SEL-ID): an intersection between teacher identity and social and emotional learning (SEL). Behav. Sci. 16:58. doi: 10.3390/bs16010058 41594999 PMC12837251

[B57] Keklik I. (2023). Internationalization of counseling: integrating the Western theories and practices into the local ways. J. Multicult. Couns. Dev. 51, 128–135. doi: 10.1002/jmcd.12252

[B58] Kiliç S. Hernandez Acton E. Zhu D. Dunsmore J. C. (2026). Parental emotion socialization and children's emotional skills and socio-emotional functioning in early childhood in Türkiye and the United States. J. Genet. Psychol. 187, 1–20. doi: 10.1080/00221325.2025.2454314 39921521

[B59] Kitzinger J. (2005). “Focus group research: using group dynamics to explore perceptions, experiences and understandings,” in Qualitative Research in Health Care, ed. I. Holloway (Maidenhead: Open University Press), 56–70.

[B60] Kounenou K. Pezirkianidis C. Blantemi M. Kalamatianos A. Kourmousi N. Kostara S. G. . (2025). Vicarious trauma and burnout among mental health professionals in Greece: the role of core self-evaluations, self-compassion, and occupational factors. Psychiatry Int. 6:100. doi: 10.3390/psychiatryint6030100

[B61] Kourmousi N. Kounenou K. Olechowska A. Szplit A. Zbróg Z. Scoda A. D. . (2025). “Nature and value of SEL in the Global North: evidence from Greece, Poland, Romania, and Türkiye,” in Social and Emotional Learning as Foundation for Future Readiness: Translating Research to Practice, eds. C. M. Park, L. Ferrari, A. D. Scoda, N. K. Ozdemir, G. Marsay, and V. S. H. Solberg (Cham: Springer Nature Switzerland), 103–143. doi: 10.1007/978-3-031-84591-8_7

[B62] Krueger R. A. Casey M. A. (2015). “Focus group interviewing,” in Handbook of Practical Program Evaluation, eds. K. E. Newcomer, H. P. Hatry, and J. S. Wholey (Hoboken, NJ: Wiley), 506–534. doi: 10.1002/9781119171386.ch20

[B63] Laverty S. M. (2003). Hermeneutic phenomenology and phenomenology: a comparison of historical and methodological considerations. Int. J. Qual. Methods 2, 21–35. doi: 10.1177/160940690300200303

[B64] Lincoln Y. S. Guba E. G. (1985). Naturalistic Inquiry. Thousand Oaks, CA: SAGE. doi: 10.1016/0147-1767(85)90062-8

[B65] Lipka J. Sharp N. Brenner B. Yanez E. Sharp F. (2005). The relevance of culturally based curriculum and instruction: the case of Nancy Sharp. J. Am. Indian Educ. 44, 31–54.

[B66] Mahfouz J. Anthony-Stevens V. (2020). Why trouble SEL? The need for cultural relevance in SEL. Occas. Pap. Ser. 2020:6. doi: 10.58295/2375-3668.1354

[B67] Ministry of National Education (MEB) (2023). K-12 beceriler çerçevesi: Türkiye bütüncül modeli [K-12 skills Framework: Türkiye Holistic Model]. Turkish.

[B68] Ministry of National Education (MEB) (2024). Türkiye Yüzyili Maarif Modeli Ögretim Programlari Ortak Metni [Türkiye Century Education Model: Curricula Common Text]. Turkish.

[B69] Minkov M. Kaasa A. (2022). Do dimensions of culture exist objectively? A validation of the revised Minkov-Hofstede model of culture. Int. Bus. Rev. 31:101974. doi: 10.1016/j.intman.2022.100971

[B70] Moustakas C. (1994). Phenomenological Research Methods. Thousand Oaks, CA: SAGE. doi: 10.4135/9781412995658

[B71] Nas T. I. Dogan A. (2020). Z kuşagindaki bireylerin kişilik özelliklerinin paternalist liderlik algilarina etkisinde örgüt kültürünün düzenleyici rolü [The moderating role of organizational culture in the effect of personality traits of individuals in generation Z on paternalistic leadership perception]. Kastamonu Üniv. IIBF Derg. 22, 30–60. doi: 10.21180/iibfdkastamonu.757746

[B72] Nowell L. S. Norris J. M. White D. E. Moules N. J. (2017). Thematic analysis: striving to meet the trustworthiness criteria. Int. J. Qual. Methods 16, 1–13. doi: 10.1177/1609406917733847

[B73] Organisation for Economic Co-operation and Development (OECD) (2021). Beyond Academic Learning: First Results From the Survey of Social and Emotional Skills. Paris: OECD Publishing.

[B74] Özen-Uyar R. Aslan D. Reinke W. M. Aktaş-Arnas Y. (2025). Training and coaching early childhood teachers to foster social, emotional, and behavioral competence of children in Turkey. Sch. Psychol. 40, 309–322. doi: 10.1037/spq0000625 38602823

[B75] Özhan M. B. Taşgin A. Kandirmaz M. (2023). K12 beceriler çerçevesi: Türkiye bütüncül modeli baglaminda sosyal duygusal ögrenme becerileri [Social emotional learning skills in the context of K12 skills framework: Türkiye holistic model]. Milli Egitim Derg. 52, 1027–1054. doi: 10.37669/milliegitim.1308964

[B76] Parrish P. Linder-VanBerschot J. (2010). Cultural dimensions of learning: addressing the challenges of multicultural instruction. Int. Rev. Res. Open Distrib. Learn. 11, 1–19. doi: 10.19173/irrodl.v11i2.809

[B77] Patton M. Q. (2014). Qualitative Research and Evaluation Methods: Integrating Theory and Practice, 4th Edn. Los Angeles, CA: SAGE.

[B78] Saldaña J. (2021). The Coding Manual for Qualitative Researchers, 4th Edn. Newcastle upon Tyne: SAGE.

[B79] Scoda A. D. Park C. M. (2025). “Cross-cultural examination of empathy,” in Social and Emotional Learning as Foundation for Future Readiness: Translating Research to Practice, eds. C. M. Park, L. Ferrari, A. D. Scoda, N. K. Ozdemir, G. Marsay, and V. S. H. Solberg (Cham: Springer Nature Switzerland), 145–161. doi: 10.1007/978-3-031-84591-8_8

[B80] Shenton A. K. (2004). Strategies for ensuring trustworthiness in qualitative research projects. Educ. Inf. 22, 63–75. doi: 10.3233/EFI-2004-22201

[B81] Sheridan S. M. Smith T. E. Moorman Kim E. Beretvas S. N. Park S. (2019). A meta-analysis of family-school interventions and children's social-emotional functioning: moderators and components of efficacy. Rev. Educ. Res. 89, 296–332. doi: 10.3102/0034654318825437

[B82] Shi J. Cheung A. C. (2024). Effective components of social emotional learning programs: a meta-analysis. J. Youth Adolesc. 53, 755–771. doi: 10.1007/s10964-024-01942-7 38280178

[B83] Smith J. A. Flowers P. Larkin M. (2009). Interpretative Phenomenological Analysis: Theory, Method and Research. Newcastle upon Tyne: SAGE.

[B84] Solberg S. Edwards A. Nyborg G. (2020). Leading for school inclusion and prevention? How school leadership teams support shy students and their teachers. Scand. J. Educ. Res. 65, 1203–1216. doi: 10.1080/00313831.2020.1788156

[B85] Suh E. Diener E. Oishi S. Triandis H. C. (1998). The shifting basis of life satisfaction judgments across cultures: emotions versus norms. J. Pers. Soc. Psychol. 74, 482–493. doi: 10.1037/0022-3514.74.2.482

[B86] Tracy S. J. (2010). Qualitative quality: eight “big-tent” criteria for excellent qualitative research. Qual. Inq. 16, 837–851. doi: 10.1177/1077800410383121

[B87] Turkish Industry and Business Association (TÜSIAD) (2019). Sosyal ve duygusal ögrenme becerileri: Yeni sanayi devriminin eşiginde iş ve yaşam yetkinliklerinin anahtari [Social and Emotional Learning Skills: The Key to Work and Life Competencies on the Threshold of the New Industrial Revolution]. Turkish. Available online at: https://tusiad.org/tr/yayinlar/raporlar/item/10450-sosyal-ve-duygusal-ogrenme-becerileri (Accessed May 10, 2025).

[B88] Uskul A. K. (1998). Individualism and Collectivism: A Social Psychological Perspective (Doctoral dissertation). Middle East Technical University, Ankara, Türkiye.

[B89] van Manen M. (2023). Phenomenology of Practice: Meaning-Giving Methods in Phenomenological Research and Writing, 2nd Edn. London: Routledge. doi: 10.4324/9781003228073

[B90] Wanless S. B. Domitrovich C. E. (2015). Readiness to implement school-based social-emotional learning interventions: using research on factors related to implementation to maximize quality. Prev. Sci. 16, 1037–1043. doi: 10.1007/s11121-015-0612-5 26466583

[B91] Weissberg R. P. Durlak J. A. Domitrovich C. E. Gullotta T. P. (2015). “Social and emotional learning: past, present, and future,” in Handbook of Social and Emotional Learning: Research and Practice, eds. J. A. Durlak, C. E. Domitrovich, R. P. Weissberg, and T. P. Gullotta (New York, NY: Guilford Press), 3–19.

[B92] Wigelsworth M. Lendrum A. Oldfield J. Scott A. ten Bokkel I. Tate K. . (2016). The impact of trial stage, developer involvement and international transferability on universal social and emotional learning programme outcomes: a meta-analysis. Camb. J. Educ. 46, 347–376. doi: 10.1080/0305764X.2016.1195791

[B93] World Health Organization (2003). Skills for Health: Skills-Based Health Education Including Life Skills: An Important Component of a Child-Friendly/Health-Promoting School. Geneva: World Health Organization. Available online at: https://iris.who.int/handle/10665/42818 (Accessed January 19, 2026).

[B94] Yildirim A. Simşek H. (2021). Sosyal bilimlerde nitel araştirma yöntemleri [Qualitative Research Methods in the Social Sciences]. Ankara: Seçkin Yayincilik. Turkish.

